# “Crown of Death”; Corona Mortis, a Common Vascular Variant in Pelvis: Identification at Routine 64-Slice CT-Angiography

**DOI:** 10.30476/BEAT.2020.84118

**Published:** 2020-07

**Authors:** Rohit Bhoil, Neeti Aggarwal, Vineet Aggarwal, Mukesh Surya, Sanjiv Sharma, Ajay Ahluwalia, Sabina Bhoil, Surya Pratap Singh, Manveer Thakur, Sidharath Sood

**Affiliations:** 1 *Department of Radiodiagnosis, IGMC Shimla, India*; 2 *Department of Orthopaedics, IGMC Shimla, India*; 3 *Department of Cardiac Anesthesia, IGMC Shimla, India*

**Keywords:** Pelvic fracture, Trauma, Haemorrhage, Vascular intervention, Embolization

## Abstract

**Objective:**

To establish the incidence of arterial corona mortis variant in angiographic studies being performed using a 64 slice CT scan machine in a series of patients.

**Methods:**

This was a prospective cross-sectional study including 100 consecutive patients undergoing routine clinically indicated, standard protocol, CT-angiography for the abdominal aorta and/or lower limbs using a 64 slice CT scanner. Patients having severe arterial insufficiency (Grade 4 stenosis on CT angiography), pelvic infections and tumours, patients with past pelvic trauma and those who had previous pelvic surgery were excluded from the study. In total 200 hemi-pelvises were evaluated for the presence or absence of corona morti.

**Results:**

Overall, we included 100 patients in this series including 67 men and 33 women with mean age of 40.1±2.3 (ranging from 22-74) years. The arterial variant was identified on thin, 0.625-mm-thick images in 24 out of 100 patients studied (unilateral in 20 patients and bilateral in 4 patients; 28 out of 200 hemipelvises evaluated, having an incidence of 14%). We found that the distance of corona mortis artery from the symphysis was significantly greater for women compared to men, both on right (*p*=0.034) and left sides (*p*=0.046).

**Conclusion:**

Corona mortis may be prospectively identified at contrast-enhanced multidetector CT especially in pelvic trauma patients and help guide subsequent endovascular embolization or surgical interventions.

## Introduction

**Fig. 1:**
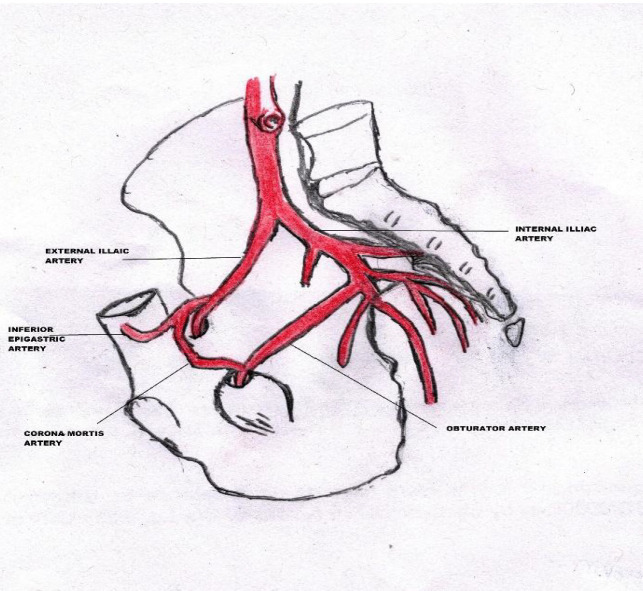
Diagrammatic representation of the arterial variant of Corona Mortis

Corona mortis is a variant vascular anastomosis in between the external iliac artery or inferior epigastric artery and obturator artery; located posterior to the superior pubic ramus, close to the quadrilateral plate of the acetabulum [1-3] (Figure 1). Also known as ‘crown of death’, the term underlines the importance of this variant, as potentially life threatening hemorrhage may occur if injured during pelvic trauma and surgeries in this region [3,4]. Knowledge of this variant vascular anastomosis is important for surgical planning, and in pelvic trauma as it is susceptible to vascular injury given its close proximity to the superior pubic ramus [3, 5]. Awareness of the typical sites of pelvic bleeding from trauma as well as the potential anatomic variations of the origin and course of bleeding arteries is also of vital importance for the interventional radiologists for the subsequent management of such patients [6-9].

We evaluated the CT angiographic studies in 100 patients for the presence of this arterial variant with the purpose to establish the incidence of corona mortis and to evaluate its anatomy (diameter of artery, its distance from symphysis pubis and relation if any with the inter-acetabular distance); so as to raise the awareness among radiologists and surgeons to the possibility of occurrence of this anastomosis in cases that involve the need to approach this area, such as in emergencies where arterial embolization is required to be done to control bleeding and in various other surgeries in this region especially orthopaedic procedures in case of pelvic fractures.

## Materials and Methods

Criteria of Selection

This was a prospective cross-sectional study carried out in the Department of Radiodiagnosis of Indira Gandhi Medical College Shimla, HP (India). The study was carried out over a period of 8 months from January 2017 to August 2017. Patients who underwent CT angiography for evaluation of the lower limb and/or pelvic arteries (mainly for acute bleeding or arterial obstruction) were included in the study.Patients having severe arterial insufficiency involving the iliac arterial system (Grade 4 stenosis on CT angiography) which potentially limited the ability to evaluate vascular opacification within small calcified vessels were excluded from the study. We have also excluded those with pelvic infections and tumours, patients with past pelvic trauma and those who had previous pelvic surgery. Written informed consent was obtained from all the patients who participated in the study. All procedures were in accordance with the ethical standards of the responsible committee on human experimentation (institutional and national) and with the Helsinki Declaration of 1975, as revised in 2000. No animals were used during the study. 

Number of Patients

Out of 146 patients considered, 46 patients were excluded from the study. The reasons for their exclusion were: Grade IV stenosis in 29 patients; pelvic tumors/masses in 7 patients; history of previous pelvic surgeries in 4 patients; past pelvic trauma in 3 patients and suspected pelvic infections in 3 patients. Overall, 100 patients who met the eligibility criteria were included in the study. Thus, a total of 200 hemipelvises were evaluated in our study for the presence or absence of (arterial) corona mortis variant.

Image Acquisition and Evaluation

**Fig. 2:**
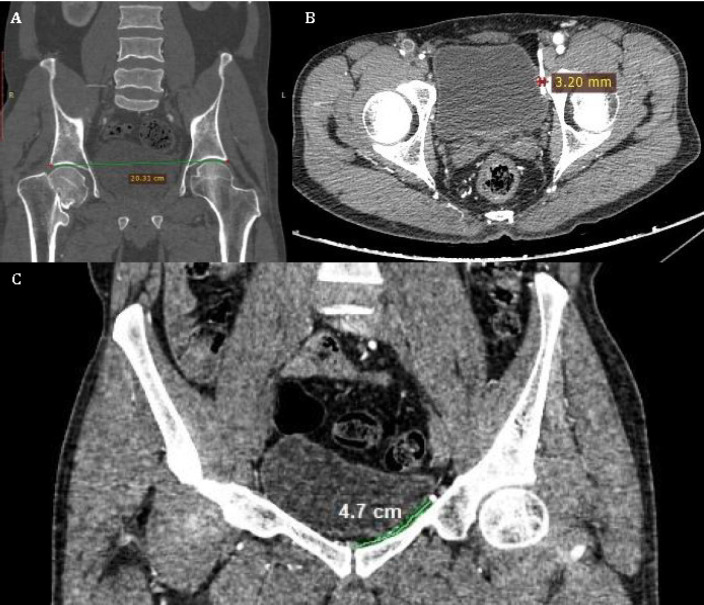
2D coronal reconstruction of CT angiographic image in a 40-year-old woman showing the measurement of the inter-acetabular distance between the right and the left acetabulum upper labarum (A); 2D axial section of CT angiographic image in a 35-year-old man showing the measurement of diameter of the vessel (left side in this case) (B); 2D coronal reconstruction of CT angiographic image (soft tissue window) in a 42-year-old woman. The distance between the symphysis and the Corona Mortis (left side in this case) is measured by using the free hand line (C).

Patients were scanned by a 64-slice CT scanner (GE LightSpeed VCT64), using 1.5ml/kg body weight of non-ionic contrast (350 mg of organic iodine; brand name: Omnipaque 350) and were scanned at a delay of 20 seconds. Slice thickness was 0.625mm acquired axially at 5mm intervals. No extra radiation/contrast was administered. Prior informed consent was taken from all the patients. Axial source images were evaluated along with multiplanar-reformatted (MPR) images using GE software. Maximum Intensity Projection (MIP) and Volume Rendering (VR) techniques were used to evaluate for the presence or absence of (arterial) corona mortis. The CT angiographic images of all eligible patients were evaluated for presence/absence of the arterial variant of corona mortis; diameter of arterial variant; and its distance from the symphysis pubis (Figure 2). For the purpose of measurement of vessel diameter, the measurement was made at a point where the artery coursed above the superior pubic ramus. In addition, the pelvic size or the inter-acetabular distance (between the right and the left acetabulum upper labrum) was also measured (Figure 2a). All the measurements were made by the same author to avoid any inter-observer variation.

Statistical Analysis

Statistical analysis was done by using Statistical Package for the Social Sciences (SPSS Inc., Chicago Illinois, USA), 16.0 for Windows. The data are presented as mean±SD and proportions as appropriate. Chi-square and Fisher’s exact tests were used to evaluate the association between the categorical variables. The independent t-test was used to compare the parametric variables with normal distribution. A two-sided *p*-value of less than 0.05 was considered statistically significant.

## Results

**Fig. 3:**
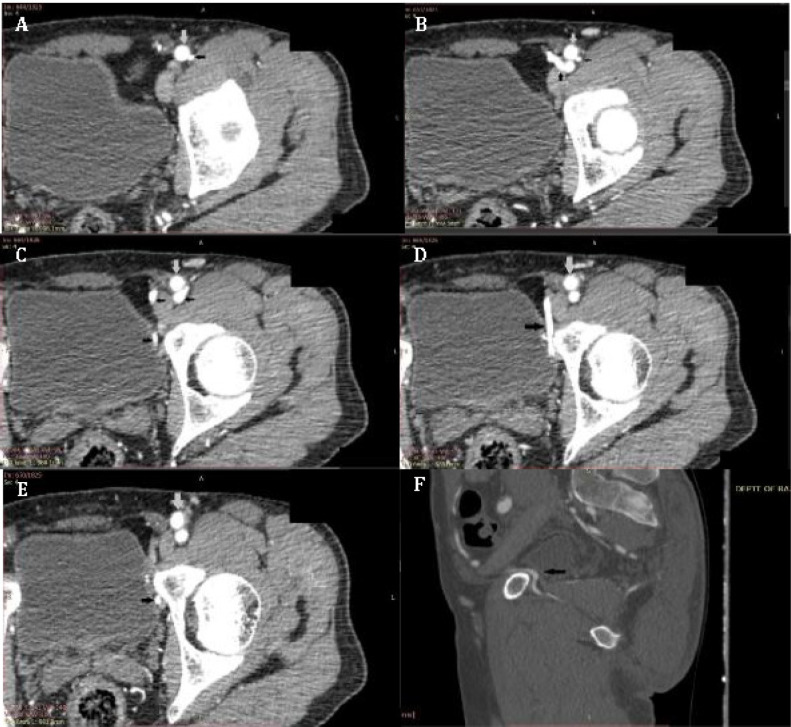
Sequential 2D axial sections (A-E; superior to inferior, soft tissue window) and (F) sagittal view (bone window) of CT angiographic images in a 35-year-oldman showing left Corona Mortis artery (black arrow; grey arrow depicts the external iliac artery).

**Fig. 4:**
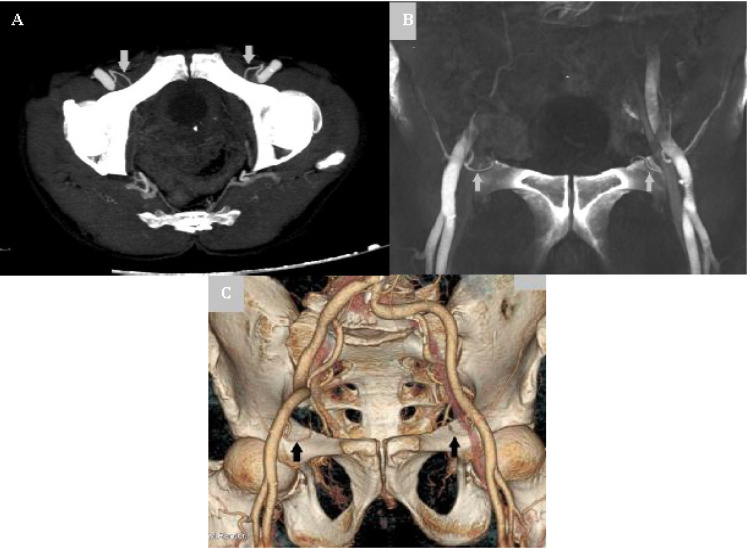
CT angiographic images in a 64-year-old woman showing the bilateral Corona Mortis arteries (arrow). (A) Axial thick MIP (Maximum intensity projection) reconstructed image; (B) coronal shaded surface rendering of the vascular CT study and (C) 3D volume rendered image

**Fig. 5:**
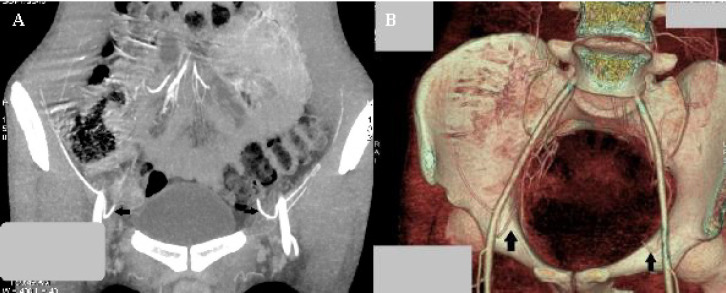
CT angiographic images in a 68-year-old man showing the bilateral Corona Mortis arteries (arrow). (A) Coronal MIP image (B) oblique 3D volume rendered image

**Fig. 6:**
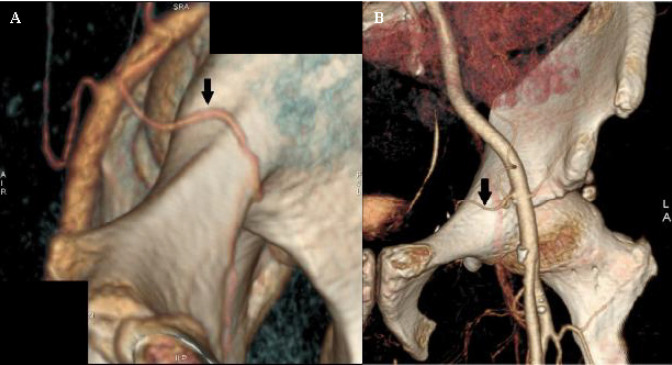
3D volume rendered images showing the Corona Mortis arteries on either side in two different patients (arrow). (A) 52-year-oldwoman with right Corona Mortis artery; (B) 47-year-old man with left Corona Mortis artery

One hundred pelvises of 67 men and 33 women with mean age of 40.1±2.3 (ranging from 22-74) years were evaluated. Data was collected from 78 lower extremity and 22 abdominal CT angiographies. The arterial variant was identified on thin, 0.625-mm-thick images (Figures 3-6) in 24 out of 100 patients studied (unilateral in 20 patients and bilateral in 4 patients; 28 out of 200 hemipelvises evaluated, having an incidence of 14%). No anastomosis was seen in 76 patients. The arterial variant was seen in 15 out of 67 (22.3%) men and 9 out of 33 (27.3%) women. This difference in gender distribution was however not significant (*p=0.261*). No significant difference was seen with respect to laterality of the variant and gender of the patient in whom the variant was detected; however, the arterial variant was seen in both sides in 4 patients (3 women and 1 man).

The mean diameter of the corona mortis artery was 2.6±0.2 mm on the right side (range: 1.7–3.0 mm) and 2.3±0.24 mm on the left side (range: 1.6–3.2 mm) (Figure 2b). No significant difference was found between the men and women in regard to diameter of corona mortis artery (*p=0.140*). None of the patients with interacetabular distance less than 188 mm showed this variant. Patients in whom the arterial variant of corona mortis was detected had a mean inter-acetabular distance of 204±8.8 mm (range: 188–220 mm) (Figure 2a).The inter-acetabular distance in women was 201±8.90 mm (range: 188–217 mm) and 208±7.73 mm (range: 192–220 mm) in men (*p=0.181)*.The mean distance from the symphysis to the corona mortis artery was 54.55±4.9 mm; (range: 42–68 mm) on the right side and 54.26 mm±5.2; (range: 40-66 mm) on the left side (*p=0.221*).This distance of corona mortis artery from the symphysis was significantly greater for women compared to men, both on right (*p=0.034*) and left sides (*p=0.046*) (Table 1) (Figure 2c).

**Table 1:** The incidence, and the mean distance (mm) of the variant from symphysis pubis in men and women

	**Women (n=33)**	**Men (n=67)**	***p*** **-value**
Incidence			
	9	15	0.261
Mean distance of the variant from symphysis pubis (Mean±SD)			
Right Side (mm)	56.20±4.52	52.90±5.20	0.034
Left Side (mm)	56.80±4.90	51.72±6.43	0.046

## Discussion

Injury to the Corona Mortis or Crown of Death is a potential source of life threatening hemorrhage in pelvic trauma, orthopaedic and surgical patients [1-5]. Knowledge of this variant anastomosis is of vital importance for surgical planning, and in pelvic trauma as it is predisposed to injury due to its posterior relation to the superior pubic ramus. It is prudent for the radiologists and surgeons to be familiar with this anatomic variant so as to avoid misdiagnosis and for safe planning of surgical procedures [4-7, 9, 10]. Our study was conducted on a 64 slice multi-detector CT which depicts a detailed anatomy of the vessels including three-dimensional (3D) images and their relationship to the bone. The aim of this study was to determine the incidence of Corona Mortis and to evaluate its anatomy in terms of its diameter, its distance from symphysis pubis and relation if any with the inter-acetabular distance.

The occurrence of corona mortis variant has been assessed in various cadaveric and CT angiographic studies [1-3]. In our study the arterial variant of corona mortis was present in 14% (28 out of 200) of the hemipelvises which is in correlation to the studies done by other researchers [10-14]. While a higher incidence up to 65% has been reported in other studies [1, 2, 4, 15-17]. This wide variation in incidence may be due to the different ethnic populations studied; different nature of study (cadaveric/angiographic); and limitations caused by the collapsed small calibre vessels in cadavers and the various dissecting approaches employed [10-12]. Amongst the studies carried on CT angiographies, the incidence of corona mortis is seen in ranging between 25-33% [1-3, 16]. Again this variation in incidence may be attributed to the different populations studied as none of these have been done on Asian or more specifically Indian population.

The average diameter of the arteries in our study was found to be 2.6 mm on right side and 2.3 mm on left side, which was in concordance with the studies carried by other researchers [1-4].

We found that corona mortis artery was located more laterally in case of females as compared to males. The distance of artery from symphysis was significantly more in females on both sides. Similar observation has been made by Steinberg*et al*., [1] however, they found that this difference in laterality was significantly more in females only on left side. It is suggested that the reason for the corona mortis artery being more lateral in females may be due to (a) less slope; (b) widely separated anterior iliac spines and (c) smaller superior aperture of the lesser pelvis in females as compared to male pelvis, due to which the artery takes a more lateral course from the symphysis pubis [1].

The average inter-acetabular distance in patients in whom corona mortis artery was detected was 206 mm (range: 188-220 mm). In our study, significant correlation was seen in between the pelvic size and the presence of the arterial variant, with none being detected in smaller pelvic sizes, i.e., inter-acetabular distance of less than 188 mm. Similar observation has been made by Steinberg, et al. [1] in their study on CT angiography, their pelvic cut-off size being 205 mm. Average inter-acetabular distance was found to be 213 mm in the study by Perandini*et al*., [3]. Smith *et al*., [2]evaluated the corona mortis artery on 16 and 64 slice CT scanners and concluded that that thin (1.25-mm) CT reconstructions demonstrated this variation much more frequently than 5-mm-thick images. We evaluated the variant on a 64 slice CT scanner with a slice thickness was 0.625 mm acquired axially at 5 mm intervals. 

Nonetheless, our study had few limitations. First, none of our patients presented with acute pelvic injury, in which case local-regional factors like fractures and hematomas, as well as a hypovolemic state may lead to decreased distension of vascular structures, thereby decreasing the likelihood of confidently identifying this variant. The correlation with actual dissection could not be made. The other limitation was that the CT angiographic images were not evaluated for the presence or absence of the venous corona mortis variant, which may also be of clinical significance. And the last limitation was a relative small study population and more studies are needed to calculate the occurrence of corona mortis both in the general population and in trauma patients.

In conclusion, the arterial variant of Corona Mortis may be prospectively identified on contrast-enhanced 64 slice CT scans which may help guide subsequent operative and endovascular embolization procedures. This variant was identified on thin, 0.625-mm-thick images in 24 out of 100 patients studied (unilateral in 20 patients and bilateral in 4 patients; 28 out of 200 hemipelvises evaluated – incidence of 14%). However, further studies in the designated population i.e. pelvic trauma patients are required to confirm this.

## Conflict of Interest: 

None declared.
